# Special Notice

**Published:** 1889-05

**Authors:** J. M. Crouse

**Affiliations:** Chairman


### Special Notice.

To the following communication the earnest attention of the readers of the Register is especially directed. It is very desirable that the questions involved in this controversy be settled at as early a date as practicable and in accord with justice and the true merits of the case. Without care and attention the plaintiff in this contest is likely to have greatly the advantage in several particulars. In the first place they have the best legal talent obtainable in the country, and that two with a long and thorough training for just such work ; it is hardly possible that the defendants can have an equal opportunity in this respect.

Second, the financial resources of the former is vastly greater than will be that of the latter without special attention and effort is given in this direction. This is only to be done by the voluntary contributions of members of the profession.

Third, the plaintiffs give a constant persevering attention and labor to the prosecution of their cause that is almost impossible to be equaled by the defendants.

In order that those who are conducting this defense shall have the means and facilities for a fair and equitable presentation of their cause and be able to bring forward, and fairly present all evidence that may serve for a right determination of the questions at issue, it would seem that every member of the profession who has the interest and welfare at heart, would promptly and with gladness comply with the request of the following note of Dr. Crouse. Don't send men to fight your cause without weapons or ammunition.

IMPORTANT DISPATCH FROM DR. CROUSE.

*Dear Doctor:*—Did you receive the circular and by-laws recently mailed to every dentist in the United States? If not, or if you have mislaid them, will gladly send you others.

Have you read the circular? If not, why not? If you have read it, have you signed and sent by-laws with membership fee, $10.00, as requested? If not, will you not do so at once? Also send us history of any cases you have or know of, as described in the circular. We have already received descriptions of many new cases which will add valuable testimony in our favor in the coming contest. Many have already joined the association but we want many more.

The amount of money required of each one is very small. The fee, however, will remain at $10.00 till a certain number is obtained, after that, it will be but just and in accord with similar organizations that those who then join shall pay a larger membership fee. We feel sure speaking from the advice of our attorneys, Messrs. Offield & Towle, that we will eventually be successful against all the claims of the Tooth Crown Company.

Many are asking if it will not cost more to settle with that company if we *should* fail? We answer no! as the rates of licenses and royalty have already been established, and such rates will be held as legal in cases of past infringements and future licenses. But we can not fail if dentists pull together.

Will you help, or hinder by holding back? Let us hear from you.

J. M. CROUSE, Chairman.

